# Parapagus dicephalus dipus tribrachius conjoined twins: a case report

**DOI:** 10.11604/pamj.2025.50.51.46382

**Published:** 2025-02-12

**Authors:** Mwaba Kopa, Lukundo Siame, Michelo Haluuma Miyoba, Hillary Sichila, Pauline Kaluba, Benson Malambo Hamooya, Collins Mukubesa, Walaza Phiri, Sydney Mulamfu, Chanda Kapoma, Sepiso Kenias Masenga

**Affiliations:** 1 Livingstone University Teaching Hospital, Department of Pediatrics, Livingstone, Zambia,; 2 Mulungushi University School of Medicine and Health Sciences, Department of Human Anatomy, Livingstone, Zambia,; 3 Livingstone University Teaching Hospital, Department of Surgery, Livingstone, Zambia,; 4 Mulungushi University School of Medicine and Health Sciences, Department of Clinical Science, Livingstone, Zambia,; 5 Mulungushi University School of Medicine and Health Sciences, Department of Public Health, Hypertension, HIV/AIDS, Nutrition, Diabetes, and Dyslipidemia (HAND) Research Group, Livingstone, Zambia,; 6 Livingstone University Teaching Hospital, Department of Radiology, Livingstone, Zambia,; 7 Mulungushi University School of Medicine and Health Sciences, Department of Physiological Science, HIV/AIDS, Nutrition, Diabetes and Dyslipidemia (HAND) Research Group, Livingstone, Zambia

**Keywords:** Conjoined twins, parapagus dicephalus, monoamniotic monochorionic, congenital anomalies, case report

## Abstract

Conjoined twins are a rare occurrence, affecting approximately 1.47 per 100,000 live births as a result of the incomplete splitting of the embryo in monochorionic monoamniotic pregnancies, after 13-14 days post-fertilization. Early prenatal diagnosis through ultrasound between 11 and 14 weeks is crucial for counseling and management decisions, including pregnancy termination or continuation with the support of a multidisciplinary team. We report a case of a 17-year-old primigravida referred in the third trimester with an ultrasound diagnosis of parapagus dicephalus dipus tribrachius conjoined twins. Delivery was by elective cesarean section, and the twins shared a single heart with three ventricles, two atria, a large atrial septal defect, and an incompetent atrioventricular valve. They died of sepsis on day 20 post-delivery. In our setting, anomaly scans during the second trimester are rarely or never performed, highlighting the need for routine early anomaly scans to enable timely intervention and improve outcomes in similar cases.

## Introduction

Conjoined twinning is a rare form of malformation that has been reported in mammals, fish, birds, reptiles, and amphibians [1]. Conjoined twins occur in about 1% of monochorionic monoamniotic pregnancies [2]. These pregnancies result from a division of the zygote occurring later than 12 to 15 days after fertilization and are associated with a high perinatal death rate [3]. The incidence of conjoined twins is estimated to be one in 100,000 births, with a higher prevalence in Africa and India with female preponderance [4]. In Zambia, the current incidence is unknown, however, a 1996 study estimated it to be between 1 in 53,000 and 1 in 52,000 live births [5]. The most common types of conjoined twins are thoracopagus, omphalopagus, pygopagus, parapagus, ischiopagus, rachipagus, and cephalopagus, with thoracopagus being the most prevalent with a low survival rate [6]. There are no known risk factors for having conjoined twins, such as age, race, parity, or heredity. A woman is not at increased risk in her next pregnancy [7]. Early prenatal diagnosis of conjoined twins, achieved through first-trimester ultrasound and potentially magnetic resonance imaging (MRI), is crucial for optimal management. This allows for comprehensive counseling of parents regarding continuing the pregnancy with planned postnatal surgery or termination [8]. In our setting, anomaly scans are rarely performed during antenatal visits. We present a case report of parapagus dicephalus dipus tribrachius conjoined twins, born to a 17-year-old primigravida. The twins were prenatally diagnosed using ultrasound in the third trimester and survived for only 20 days after delivery.

## Patient and observation

**Patient information:** a married 17-year-old primigravida was referred to our facility in the third trimester around 36 weeks of gestation following her first antenatal ultrasound that revealed parapagus dicephalus, conjoined twins. The patient had an unremarkable antenatal history without a family history of multiple gestation or any other medical history. The patient was scheduled for an elective cesarean section which was performed at 38 weeks of gestation with an outcome of live conjoined twins with Apgar scores of 5 and 6 at 1 and 5 minutes, respectively, and a combined weight of 3.2 kg. Immediate post-delivery management involved stabilization and transfer to the Neonatal Intensive Care Unit (NICU).

**Figure 1:**
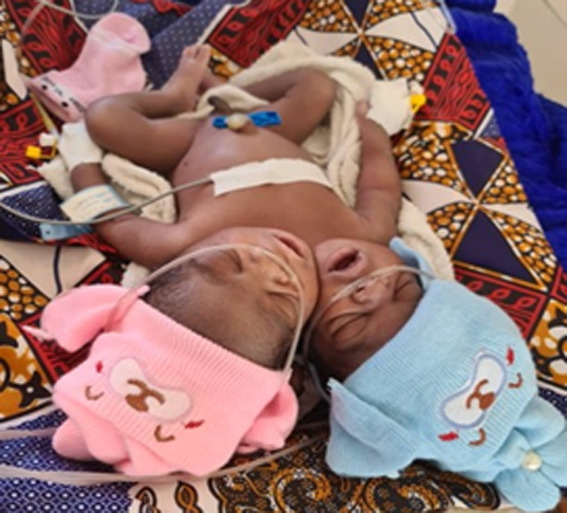
parapagus dicephalus dipus tribrachius twins: the image highlights the shared body structure, including two heads, three arms, and two lower limbs, consistent with the parapagus dicephalus dipus tribrachius configuration

**Clinical findings:** postnatal physical examination revealed that the twins had two heads; a chest that was symmetrical with two equidistant nipples; the abdomen was normal in contour and the umbilical cord contained one vein and a single artery; two well-formed arms, a rudimentary arm in between the two heads, and two well-formed legs. Examination of the back showed two spines with no kyphosis or scoliosis. The twins shared a single set of well-formed male external genitalia, with a scrotum without testes, and a single anus (Figure 1). Temperature of 36.4 degrees Celsius, oxygen saturation (SpO_2_) of 91%, pulse of 161 beats per minute, and random blood glucose of 4.8 mmol/L.

**Timeline of the current episode:** a 17-year-old primigravida with an uneventful early pregnancy underwent her first antenatal ultrasound at 36 weeks, conjoined twins. At 38 weeks, an elective cesarean section delivered live parapagus dicephalus dipus tribrachius twins with Apgar scores of 5 and 6. They were stabilized and admitted to the NICU, where examinations confirmed their condition and identified shared organs and cardiac anomalies. Despite supportive care, including oxygen therapy, nasogastric feeding, cardiac medications, and antibiotics for *Klebsiella pneumoniae* sepsis, the twins succumbed to complications from sepsis and cardiac issues on day 20 post-delivery.

**Figure 2:**
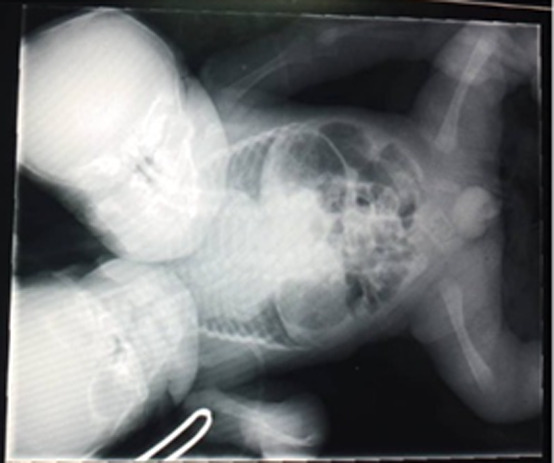
X-ray of parapagus dicephalus dipus tribrachius twins: the radiograph illustrates the shared skeletal anatomy, including a single thoracic cavity and pelvis, with distinct cranial structures and additional details of organ arrangement

**Diagnostic assessment:** X-ray revealed two separate vertebral columns joining at the pelvic brim (Figure 2). The upper and lower limbs appeared normal. Abdominal ultrasound showed an enlarged liver, single gallbladder, single spleen, single left kidney, and two stomach bubbles. The diaphragm was intact with two spinal cords extending from the cervical to coccygeal regions. Echocardiography indicated a single heart with three ventricles, two atria, a large atrial septal defect, and an incompetent atrioventricular valve on the right. Cranial ultrasound demonstrated normal brain structures in both heads. The laboratory results revealed a normal complete blood count: white blood cell count of 10.20 x 10^9^/L, red blood cell count of 3.82 x 10^9^/L, hemoglobin level of 12.5 g/dL, mean corpuscular volume (MCV) of 102.0 fL, and platelet count of 229 x 10^9^/L. The kidney function test showed a creatinine level of 97.3 µmol/L and a urea level of 5.7 mmol/L. Additionally, the blood culture isolated *Klebsiella pneumoniae*.

**Diagnosis:** parapagus dicephalus dipus tribrachius conjoined twins with univentricular heart, atrial septal defect (ASD), atrioventricular valve regurgitation, and early onset neonatal sepsis due to *Klebsiella pneumoniae*.

**Therapeutic interventions:** the twins were fed expressed breast milk via a nasogastric tube and received oxygen therapy at 2L/min via nasal prongs. Due to the cardiac anomaly, they were administered spironolactone (2.5 mg PO BD) and hydrochlorothiazide (3 mg PO BD). Temperature spikes were noted from the first day of life and diagnosed with early onset neonatal sepsis due to *Klebsiella pneumonia* and were managed with meropenem (105mg IV TDS).

**Follow-up and outcome of interventions:** the patients were scheduled for transfer to a higher center for further consultation with pediatric surgeons, but the twins died on day 20 before the transfer.

**Patient perspective:** “We wanted the babies to survive and have a normal life. It´s unfortunate they died”.

**Informed consent:** verbal and written informed consent was obtained from the patient´s guardian for the publication of this case report and accompanying images. Ethical approval from the Mulungushi University ethics committee (Ref. No: SMHS-MU2-2024-255) was obtained on 29^th^ May 2024.

## Discussion

Conjoined twins are an extremely rare form of monozygotic twinning globally with an estimated prevalence of 1.47 per 100,000 live births [6]. Conjoined twins are classified by their point of connection. The most prevalent type is thoracopagus (joined at the chest), followed by omphalopagus (abdomen), pygopagus (sacrum), ischiopagus (pelvis), craniopagus (skull), and rachipagus (back) [9]. A rarer type, parapagus, occurs in 11% to 13% of cases and involves fusion at the pelvis and abdomen with separate heads and rudimentary arms in our case report [6]. The cause remains a mystery, however, one theory of fission is mostly a result of an incomplete zygote split that occurs after day 13 [3]. The fusion theory suggests that two separate fertilized eggs are completely separate, but their stem cells (cells with the potential to develop into different types of cells) migrate and fuse, leading to conjoined twins [3,9].

Early diagnosis of conjoined twins is important for informed management decisions and counseling of the parents [8]. Diagnosis of conjoined twins can occur as early as 8 weeks of gestation, although high-resolution ultrasound examinations and magnetic resonance imaging (MRI) between 11 and 14 weeks are more sensitive [8]. Key diagnostic signs include an inability to distinguish separate fetal bodies, two heads in the same plane, abnormal cervical spine positioning, and a lack of fetal movement despite maternal activity or manual manipulation [8]. In our case report, post-delivery ultrasound showed that the twins were joined at the head and had one enlarged liver, one gallbladder, one spleen, one left kidney, two stomach bubbles, two spinal cords, and a single heart with three ventricles, two atria, a large atrial septal defect, and an incompetent atrioventricular valve on the right.

Management of conjoined twins requires a multidisciplinary approach if the pregnancy is allowed to proceed to term [10]. In our case, obstetricians performed the cesarean section and offered prenatal diagnosis and counseling, while surgeons played a crucial role in deciding if separation was viable. Three categories guide this decision: category I (no separation), where extensive cardiac fusion makes it impossible to construct a single-functioning heart, as in our case; category II (emergency separation), required when the death or imminent death of one twin threatens the life of the other, or when a life-threatening anomaly demands urgent intervention; and category III (planned separation), undertaken when the twins are stable enough for detailed imaging and precise mapping to guide the procedure [10]. This multidisciplinary approach is vital for planning the delivery, reviewing the viability of surgical treatments, and assuring optimal postnatal care [10]. Additionally, decisions on the continuation of the pregnancy or its termination should involve detailed discussions with the parents, discussing all associated risks and potential outcomes [8]. The prognosis for conjoined twins is generally poor, with an overall survival rate of approximately 25% [9]. Parapagus twins, a rarer type involving fusion at the pelvis and abdomen, often have a poorer prognosis due to shared vital organs such as the heart, liver, and intestines [6]. Surgical separation is rarely attempted in such cases because of the complexity of shared anatomy [10]. In the present case, parapagus twins survived only 20 days due to significant organ anomalies. Early prenatal diagnosis and planned multidisciplinary care can improve outcomes in selected cases [10].

## Conclusion

This case report presents a case of parapagus dicephalus dipus tribrachius conjoined twins of a patient who presented late in pregnancy at 36 weeks of gestation for her first antenatal ultrasound, which showed the anomaly. This case highlights the need to include and enhance routine early antenatal care in our setting to enable timely diagnosis of congenital anomalies, as late presentation limits management options and decision-making opportunities for both the family and healthcare providers.
